# Leaky barriers to gene sharing between locally co-existing coagulase-negative *Staphylococcus* species

**DOI:** 10.1038/s42003-023-04877-0

**Published:** 2023-05-03

**Authors:** Odion O. Ikhimiukor, Stephanie S. R. Souza, Michael M. Marcovici, Griffin J. Nye, Robert Gibson, Cheryl P. Andam

**Affiliations:** 1 Department of Biological Sciences, University at Albany, State University of New York, Albany, NY USA; 2 Department of Molecular, Cellular and Biomedical Sciences, University of New Hampshire, Durham, NH USA; 3 New Hampshire Veterinary Diagnostic Laboratory, Durham, NH USA; 4 Present Address: The Jackson Laboratory, 600 Main Street, Bar Harbor, ME USA

**Keywords:** Pathogens, Molecular evolution

## Abstract

Coagulase-negative *Staphylococcus* (CoNS) are opportunistic pathogens implicated in many human and animal infections. The evolutionary history of CoNS remains obscure because of the historical lack of recognition for their clinical importance and poor taxonomic sampling. Here, we sequenced the genomes of 191 CoNS isolates representing 15 species sampled from diseased animals diagnosed in a veterinary diagnostic laboratory. We found that CoNS are important reservoirs of diverse phages, plasmids and mobilizable genes encoding antimicrobial resistance, heavy metal resistance, and virulence. Frequent exchange of DNA between certain donor-recipient partners suggests that specific lineages act as hubs of gene sharing. We also detected frequent recombination between CoNS regardless of their animal host species, indicating that ecological barriers to horizontal gene transfer can be surmounted in co-circulating lineages. Our findings reveal frequent but structured patterns of transfer that exist within and between CoNS species, which are driven by their overlapping ecology and geographical proximity.

## Abstract (web summary)

Overlapping ecology and geographical proximity drives horizontal gene transfer and thus the evolution of opportunistic pathogens from the Coagulase-negative Staphylococcus group, as revealed by comparative genomics.

## Introduction

*Staphylococcus* species are classified as either coagulase-negative or coagulase-positive based on the ability of the coagulase enzyme to stimulate clot formation in plasma^1^. Coagulase-negative *Staphylococcus* (CoNS) species are ubiquitous members of the human microflora, frequently colonizing the skin and mucosal surfaces^2^. They have been historically considered non- or less pathogenic compared to the more prominent coagulase-positive *Staphylococcus aureus*^1^. However, many CoNS species are now considered as major opportunistic pathogens that cause serious infections in humans and animals. They have been implicated in bloodstream infections^3^, laryngological diseases^4^, skin and soft tissue infections^5^, infections of the central nervous system^6^, and infections from indwelling/implanted medical devices (e.g., catheters, prosthetic implants)^7,8^. Approximately 8% of all cases involving valve endocarditis are associated with CoNS species, with 25% of cases resulting to mortality^9^. Increasing prevalence of multidrug-resistant CoNS and resistance to newer antimicrobial agents have compounded the burden of CoNS infections and limits available treatment options^10,11^.

The evolutionary history of CoNS has not been well characterized because of the historical lack of recognition for their clinical importance and poor taxonomic sampling^1,12^. They are also often misclassified or overlooked in sampling efforts. Among *Staphylococcus* species, most investigations of evolutionary processes and genetic diversity have focused on the more well-known *S. aureus* (e.g., refs. ^13–15^). However, there is growing evidence that recombination has played a major role in CoNS evolution^16–18^. Homologous recombination involves the incorporation of exogenous DNA segment that exhibits high sequence similarity with existing DNA segment in the chromosome^19^. It can lead to either allelic replacements or the addition of new DNA segments when two flanking regions of high DNA similarity initiate the recombination process and mediate successful horizontal gene transfer^20^. We recently reported that rates of recombination rates varied among ten CoNS species in a global dataset^18^. Despite the significance of these findings, our previous inferences were derived from genome sequences from highly variable ecological sources (humans, animals, food products, environment) and geographical origins (seven continents and the International Space Station)^18^, which can greatly influence patterns and frequencies of recombination.

We hypothesized that CoNS with overlapping ecologies within a defined geographical space will exhibit frequent but structured patterns of horizontal gene transfer through homologous recombination and mobile genetic elements. Here, we sequenced the genomes of 191 CoNS isolates representing 15 species from animals diagnosed with disease in a veterinary diagnostic laboratory. We found that CoNS are an important reservoir of diverse phages, plasmids, and mobilizable genes conferring resistance to 13 antimicrobial classes. We identified frequently recombining genes, variation in accessory genomes, and patterns of DNA donation and receipt within and between CoNS species. Our findings provide a firm understanding of CoNS evolution and inform surveillance efforts and design of future treatment strategies for CoNS infections.

## Results

### Species and genomic diversity of CoNS from diseased animals

**Fig. 1:**
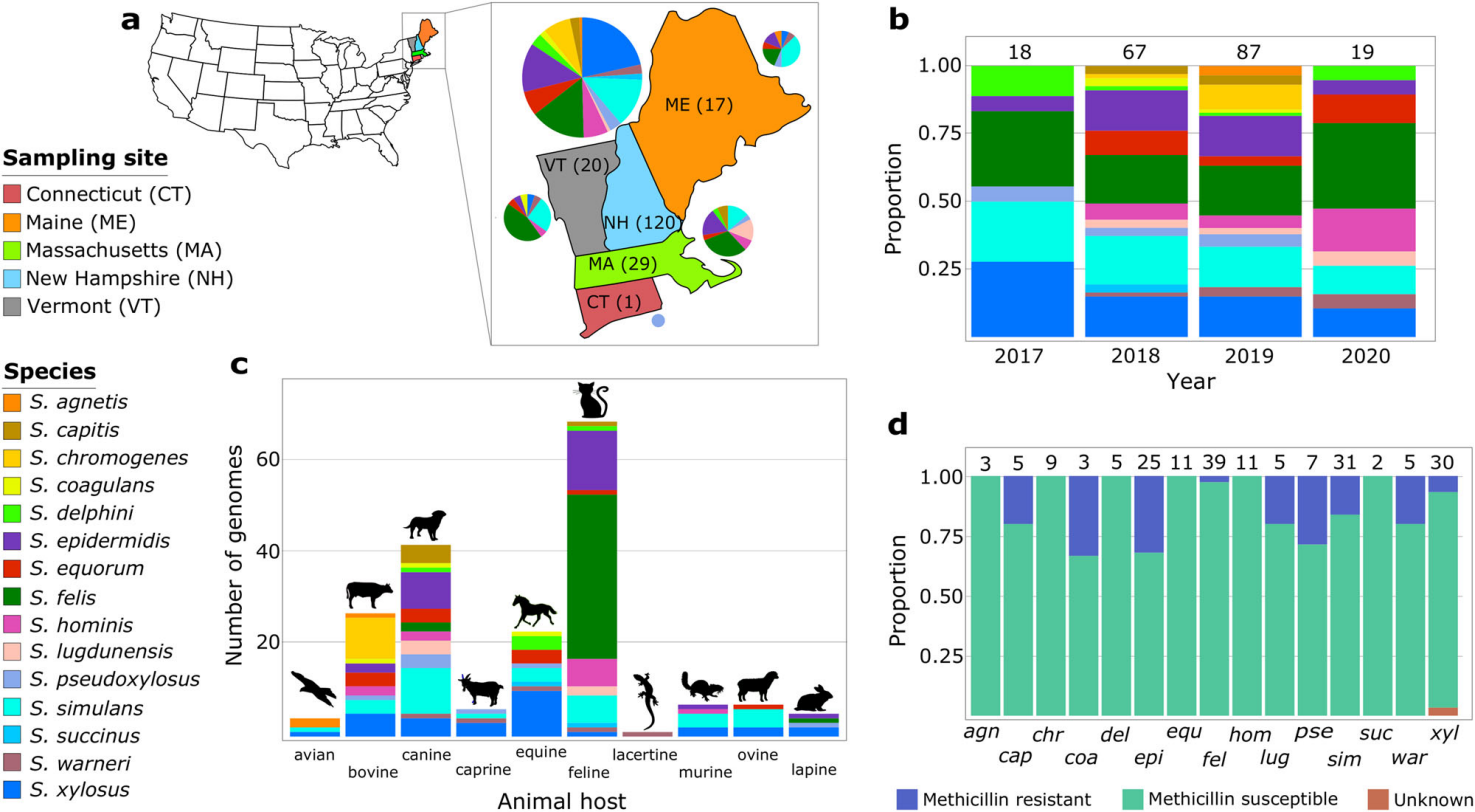
Sampling characteristics of the 191 CoNS genomes. **a** Geographical sources of CoNS species. Pie charts show the proportion of CoNS species per state. Numbers in parenthesis indicate the number of CoNS genomes per state. Two isolates came from animals from Alabama but were diagnosed in New England are not shown here. **b** Distribution of CoNS isolates and species per year of sampling. **c** Distribution and frequency of CoNS isolates per animal host. **d** Proportion of methicillin-resistant and methicillin-susceptible isolates per CoNS species determined by in vitro testing. Numbers on top of bars in panels **b** and **c** indicate the number of genomes in that group. For visual clarity in panel **d**, only the first three letters of the species name are shown.

A total of 191 CoNS isolates from diseased animals were retrieved from specimens sent to the New Hampshire Veterinary Diagnostic Laboratory (NHVDL) in the New England region of the United States (Fig. 1a, Supplementary Data 1). The isolates were sampled from 2017 to 2020 (Fig. 1b) from 10 animal species (Fig. 1c). Most isolates were obtained from cats (*n* = 69), dogs (*n* = 42), cows (*n* = 27), and horses (*n* = 23). Canine and feline hosts harbored the highest species diversity of CoNS, with 12 and 11 CoNS species, respectively (Fig. 1c). However, this is likely due to sampling bias in our dataset that heavily favored these two animal hosts. In vitro screening revealed the presence of 22 methicillin-resistant isolates, representing 11.52% of the dataset (Fig. 1d).

**Fig. 2:**
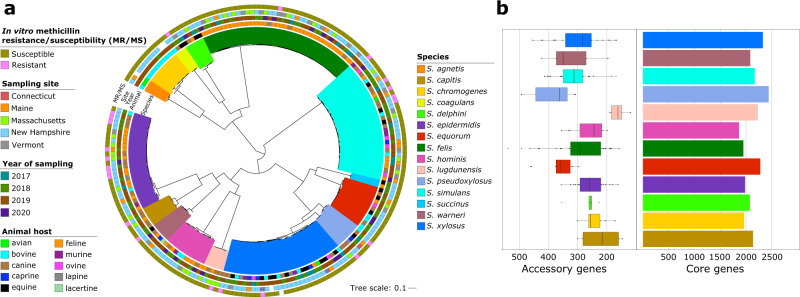
Phylogenetic relationships and pan-genome characteristics of the 191 CoNS genomes. **a** Midpoint-rooted maximum likelihood phylogenetic tree based on sequence alignment of 1317 core genes. Tree scale represents the number of nucleotide substitutions per site. Outer rings show the in vitro methicillin susceptibility, sampling site (U.S. state), year of sampling, and animal host (outer to inner rings). Branches are colored according to species. **b** Sizes of core genomes and accessory genomes per CoNS species. Only the 12 most common species are shown. (Left) Box plots showing the number of accessory genes at 25th, 50th, and 75th percentile. Black dots show the number of accessory genes outside the interquartile range. (Right) Bar plots showing the number of core genes in each CoNS species.

Whole genome sequencing of the isolates yielded high-quality genomes (Supplementary Data 1, Supplementary Fig. 1). Genome sizes ranged from 2.17 Mbp to 3.13 Mbp (mean = 2.5 Mbp). The mean number of contigs was 34 (range = 11–70), while the mean N50 was 329,792 bp (range = 70,109–1,390,089 bp). We assessed species delineation using the average nucleotide identity (ANI) of all orthologous genes shared between any two genomes with a 95% cutoff to define a species^21^ (Supplementary Fig. 2 and Supplementary Data 2). We also built a maximum likelihood phylogenetic tree based on 735,124 single nucleotide polymorphisms (SNPs) detected in the 1.27 Mbp alignment of 1317 core genes (Fig. 2a). We identified a total of 15 CoNS species. The most common species were *Staphylococcus felis* (*n* = 39 genomes), *Staphylococcus simulans* (*n* = 31), *Staphylococcus xylosus* (*n* = 30), and *Staphylococcus epidermidis* (*n* = 25), whereas the least common species were *Staphylococcus succinus* (*n* = 2), *Staphylococcus agnetis* (*n* = 3), and *Staphylococcus coagulans* (*n* = 3). However, we also detected 54 genomes that showed taxonomic discrepancies between the initial species identification using matrix-assisted laser desorption/ionization time-of-flight mass spectrometry (MALDI-TOF MS) and the genome-based species delineation. Nonetheless, within each named CoNS species, isolates from different geographical sources, animal hosts, or year of sampling were intermingled within the phylogeny.

For those species represented by more than three genomes, we estimated their core and accessory genome variation (Fig. 2b). Species with the highest number of core genes were *Staphylococcus pseudoxylosus* (*n* = 2433 genes), *S. xylosus* (*n* = 2322) and *Staphylococcus equorum* (*n* = 2271), whereas *Staphylococcus hominis* (*n* = 1860), *S. felis* (*n* = 1944) and *Staphylococcus chromogenes* had the lowest number of core genes (*n* = 1958). The number of accessory genes were highly variable across different species. *Staphylococcus lugdunensis* had the least number of accessory genes (range = 118–189; median = 161), whereas accessory gene content was highest in *S. pseudoxylosus* (range = 308–494; median = 363), *S. equorum* (range = 296–463; median = 359), *Staphylococcus warneri* (range = 196–424; median = 350) and *S. simulans* (range = 168–415; median = 313). Furthermore, core genome SNPs computed against the ANI values revealed a strong negative correlation (*R* = −0.97 to −1, *p* < 0.001) between genome pairs across the different CoNS species (Supplementary Fig. 3).

### Genetic determinants of antimicrobial resistance (AMR) and virulence in CoNS

**Fig. 3:**
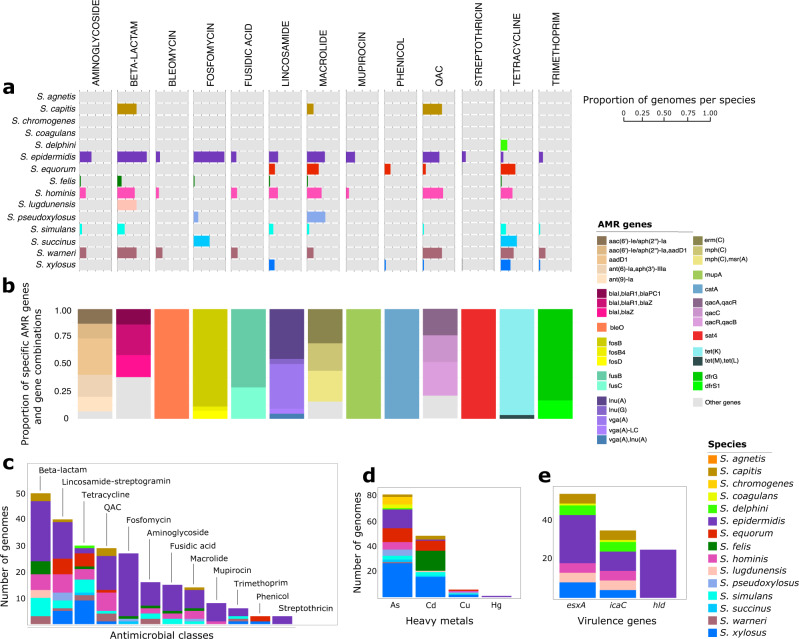
Phylogenetic distribution of AMR, heavy metal resistance, and virulence genes in CoNS. **a** Proportion of genomes within each species that carry at least one gene that confers resistance to each of the 13 antimicrobial classes. Only the 12 most common CoNS species are shown. QAC—quaternary ammonium compounds. **b** Proportion of the most frequent AMR genes and gene combinations per antimicrobial class. Each bar stack corresponds to the antimicrobial class listed above panel (**a**). **c** Number of CoNS genomes and species harboring at least one resistance gene from each antimicrobial class. **d** Number of CoNS genomes and species harboring heavy metal resistance genes. **e** Number of CoNS genomes and species harboring virulence genes.

We carried out an in silico screening of all genomes to identify acquired genes that contribute to AMR and virulence. We detected a total of 43 distinct genes that confer resistance to 13 classes of antimicrobial compounds (Fig. 3 and Supplementary Data 3). Among all species, *S. epidermidis* harbored AMR genes associated with 12 of the 13 antimicrobial classes (Fig. 3a). Other species that carried high number of AMR genes per antimicrobial class were *S. hominis*, *S. simulans*, and *S. warneri*, with at least one genome of each species carrying AMR genes associated with nine, eight, and seven antimicrobial classes, respectively. We did not detect any AMR genes in *S. agnetis*, *S. chromogenes*, and *S. coagulans*. The CoNS community has a diverse assemblage of specific AMR genes and combinations of AMR genes (Fig. 3b). The most frequently detected AMR genes were *blaI* (*n* = present in 50 genomes from seven species), *blaZ* (*n* = 42 genomes from six species), *tetK* (*n* = 29 genomes from nine species), and *fosB* (*n* = 24 genomes of *S. epidermidis*). The highest number of AMR genes per genome were detected in two *S. epidermidis* isolates from dogs (*n* = 14 and *n* = 13 AMR genes) and in *S. warneri* (*n* = 13 AMR genes) isolated from a horse. Overall, genes associated with beta-lactam resistance were most prevalent in the CoNS dataset (Fig. 3c).

We also sought to determine the presence of the *mecA* gene, which encodes an extra penicillin-binding protein that has low affinity to virtually all beta-lactam antibiotics^22^. The *mecA* gene is carried by the mobile chromosomal cassette SCC*mec*^23^. Results revealed the presence of the *mecA* gene in 18 genomes (Supplementary Fig. 4 and Supplementary Data 4). Among the 14 structurally distinct SCC*mec* types (I–XIV) that have been described^23^, we found that the *mecA* gene was carried in SCC*mec* types I (subtype Ia), III (subtype IIIa), and IV (subtypes IVa, IVc, Ivd, Ivg, Ivh) in genomes of *S. epidermidis* (*n* = 15), *S. hominis* (*n* = 1), *S. lugdunensis* (*n* = 1), and *S. simulans* (*n* = 1), and *S. warneri* (*n* = 1). We did not detect the *mecC* gene, which is a divergent form of *mecA* and also mediates beta-lactam resistance^24^. We found a slight discrepancy between the results of the in silico detection of the *mecA* gene and in vitro phenotypic testing for methicillin resistance. There were 12 isolates (*S. capitis* = 1, *S. coagulans* = 1, *S. felis* = 1, *S. simulans* = 5, *S. pseudoxylosus* = 2*, S. xylosus* = 2) whose genomes did not contain *mecA* but were phenotypically tested as methicillin resistant. On the other hand, eight isolates contained *mecA*, but were phenotypically tested as methicillin susceptible. These conflicting results may be caused by sequencing and laboratory errors, but may also suggest the absence of transcriptional regulatory genes for *mecA* for these isolates or the existence of alternative mechanisms of methicillin resistance that are yet to be discovered.

We also screened the CoNS genomes for the presence of genes associated with heavy metal resistance. We detected eight genes associated with resistance to arsenic (*arsB*, *arsC*, *arsR*), cadmium (*cadD*, *cadC*), copper (*mco*), and mercury (*merA, merT*) (Fig. 3d and Supplementary Data 3). Except for *S. agnetis*, at least one member of each CoNS species harbored a heavy metal resistance gene. Most of these genes were carried by isolates of *S. equorum, S. pseudoxylosus*, and *S. xylosus*, which are closely related in the phylogeny. A total of 83 genomes representing 43.23% of the dataset carried at least one gene conferring resistance to arsenic. These *ars*-carrying genomes represented 12 CoNS species. A total of 49, six, and one genome(s) carried at least one gene associated with resistance to cadmium, copper, and mercury, respectively.

Three virulence genes were detected in the CoNS genomes. The genes *esxA*, *icaC*, and *hld* were present in 14, 30, and 25 genomes, respectively (Fig. 3e). The *icaC* gene is part of the *ica* operon which plays an essential role in mediating the formation of biofilm and has been implicated in therapeutic failure of staphylococcal device-related infections^25^. The *esxA* gene plays a vital role in the establishment of infection in hosts by facilitating interactions with host receptor proteins^26^. Both *esxA* and *icaC* were detected in genomes from seven species (*S. agnetis, S. capitis, S. coagulans, S. delphini, S. epidermidis, S. lugdunensis, S. xylosus*). The *hld* gene is implicated in the lysis of erythrocyte cells^27^ and were exclusively detected in all members of *S. epidermidis* in our dataset.

### Lineage shapes the accessory genomes of CoNS

We carried out a network analysis based on the Jaccard similarity of accessory gene content to determine if similarity in accessory gene content between any two genomes is influenced by the genetic background (i.e., higher similarity of accessory genomes within each *Staphylococcus* species regardless of animal source) or by animal host (i.e., higher similarity of accessory genomes within each animal host regardless of *Staphylococcus* species) (Supplementary Fig. 5). Our results confirmed the former, showing distinct clustering of genomes by species (top panel) regardless of the identity of the host species (bottom panel). There was no observable clustering of the bacterial accessory genomes when mapped by their host of isolation. For example, the largest cluster representing *S. felis* consisted of isolates from feline, canine, and lapine hosts. This result shows that sharing of accessory genes between CoNS lineages can traverse species boundaries of their animal hosts. We also observed two clusters that contained a mix of two species (*S. pseudoxylosus* and *S. xylosus*; *S. agnetis* and *S. chromogenes*). These two species pairs are sister clades in the core genome phylogenetic tree (Fig. 2a), which may explain the close similarities of their accessory genomes.

### Phage and plasmid diversity in CoNS

**Fig. 4:**
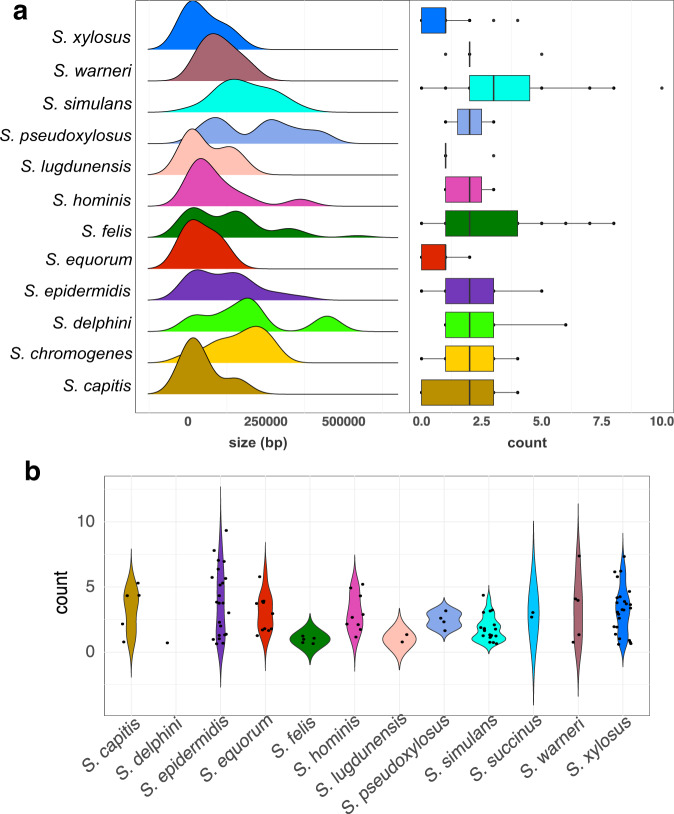
Phage and plasmid replicon diversity in CoNS. **a** Density plot showing the distribution of concatenated phage sequences sizes per genome per species of CoNS. The boxplot on the right shows the number of phages per genome per species at 25th, 50th, and 75th percentile. Black dots show the number of accessory genes outside the interquartile range. **b** Violin jitter plot showing number of plasmid replicons detected in each genome per species. Black dots represent the genomes that comprise each species. For both panels, only the 12 most common species are shown.

Mobile genetic elements such as phages and plasmids are known to influence the structure and evolution of bacterial genomes^28^. We therefore investigated the diversity of phages and plasmid replicons in CoNS (Fig. 4a and Supplementary Data 5). Phage DNA was detected in 83.26% (*n* = 159) of the CoNS isolates. The number of phages per genome ranged from 0 to 12 (median = 2). *S. agnetis* isolate 4727_3A_S125 from a bird contained the highest number of phages (*n* = 12), while the genomes of *S. simulans* isolate 7856_8A from a cow and *S. agnetis* isolate 6361e_S103 from a bird possessed 10 and nine phages, respectively (Fig. 4a left panel, Supplementary Data 5). Staphylococcal phages are highly diverse and can reach up to 152 Kb in size, occupying a substantial portion of the bacterial host chromosome^29^. In our study, the total combined genome size of phages was largest in *S. felis* isolate1657A_S114 from a cat with 545 Kbp representing 20.83 % of its whole genome. This was followed by *S. delphini* isolate 66A_S182 from a dog (446 Kbp) and *S. pseudoxylosus* isolate 3581D_S_S66 from a goat (423 Kbp), representing 16.76% and 13.54% of their whole genomes, respectively. However, we do note that there was substantial variation in the total length of phage DNA and number of phages per genome within each species.

We further screened the concatenated phage sequences for the presence of antimicrobial and virulence genes (Supplementary Data 5). The *fusB* gene was detected in *S. epidermidis* (isolates from rodent = 1 and cat = 1), whereas *mphC*, *msrA*, and *qacA* genes were detected in an *S. epidermidis* isolate from a cat. Furthermore, *blaZ* and *erm44* were detected in *S. pseudoxylosus* and *S. warneri*, respectively. Copper resistance gene *cadD* and virulence resistance gene *hld* were detected in phage sequences from *S. epidermidis* isolates 9306A_S137 and 2712_A_S183 from cats.

Analysis of short reads does not provide reliable information on plasmid genomes and associated gene content. Nonetheless, we used the presence of the gene that codes for the plasmid replicon initiator protein (*rep*) as an indication of a plasmid in the bacterial genome^30^ (Supplementary Data 6). We detected a range of 1–9 *rep* genes per genome, with *S. epidermidis* isolate 4088_3C from a cow carrying the highest number. Members of *S. epidermidis*, *S. succunis,* and *S. warneri* had at least one plasmid replicon detected in their genomes (Fig. 4b). In contrast, no plasmid replicons were detected in members of *S. chromogenes* and *S. coagulans*, while fewer representatives from species such as *S. delphini* (*n* = 1/5), *S. felis* (*n* = 5/39), and *S. pseudoxylosus* (*n* = 4/7) contain plasmid replicons in their genomes. Similar to phages, we observed considerable variation in the number and types of *rep* genes among members of each CoNS species.

### Some lineages have acquired more recombined DNA than others

**Fig. 5:**
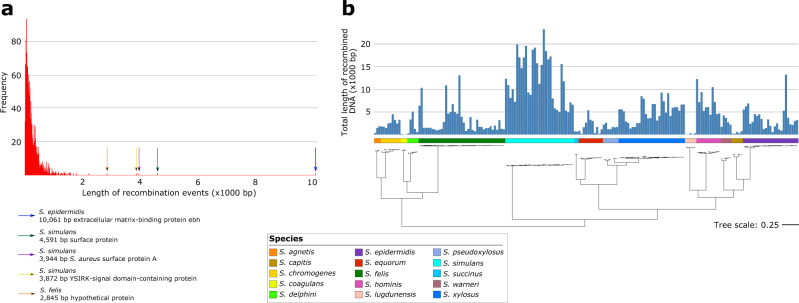
Frequently recombining CoNS lineages. **a** Histogram showing the distribution of fragment sizes of recombined DNA in CoNS. The longest recombination events are highlighted with arrows. **b** Total length of recombined DNA per genome. The tree is identical to that in Fig. 2a.

We next sought to determine the impact of homologous recombination in the core and shared accessory genes of CoNS. Using fastGEAR^31^, we identified a total of 1882 recombination events that occurred within and between species (Supplementary Data 7). The lengths of the recombined DNA segments exhibited an exponential distribution and consists of frequent short recombination events (<500 bp) and rarer large recombination events (Fig. 5a and Supplementary Data 8). The median length of recombined fragments is 242 bp. The largest recombination event was 10,061 bp long and was detected in *S. epidermidis*. It contained the *ebh* gene encoding the extracellular matrix-binding protein. Three large recombination events were also detected in *S. simulan*s isolates: a 4591 bp DNA fragment that encompassed the *pls* surface protein, a 3944 bp that contained that *S. aureus* surface protein A, and a 3872 bp DNA that contained the YSIRK-signal domain-containing protein. Summing up the total length of recombined DNA per isolate, we found that *S. simulans* isolates were the most frequent recipients of recombined DNA (mean = 12,434 bp; range = 4902–23,217 bp) (Fig. 5b). Other frequently recombining species were *S. hominis* (mean = 7022 bp and range = 4378–12,243 bp), and *S. xylosus* (mean = 5041 bp and range = 1335–9233 bp). For *S. simulans*, *S. hominis*, and *S. xylosus*, these recombination events were detected in 446, 148, 198 genes and which represent 11.53%, 5.04%, and 4.73% of each species’ pan-genome, respectively. Within the same species, the impact of recombination varied among genomes. Certain isolates of *S. epidermidis* and *S. felis* had experienced more frequent recombination compared to other isolates within the species. These included isolate 6101a_S160 (*S. epidermidis* from a cat) with 13,191 bp of total recombined DNA whereas isolates 4602_AN3_S151 and 1405b_S102 (both *S. felis* from cats) have a total of 13,191 bp and 10,845 bp recombined DNA, respectively. In contrast, the species whose genomes contained the shortest total length of recombined DNA were *S. capitis* (mean = 445 bp and range = 751–190 bp), *S. coagulans* (mean = 59 bp and range = 29–120 bp), and *S. lugdunensis* (mean = 115 bp, range = 16–284 bp). Overall, these results that certain lineages have had a history of more frequent recombination than other lineages.

### Frequent recombination in some donor-recipient partners

**Fig. 6:**
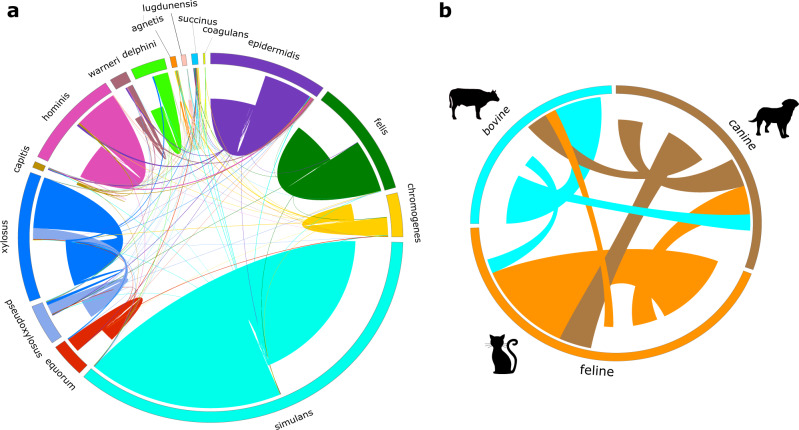
Intra- and inter-species recombination in 191 CoNS genomes. **a** Chord diagram showing the recombination events within and between members of each CoNS species. Each colored block in the outer ring represents a CoNS species, while the length of block represents the number of genomes for that species in which recombination was detected. **b** Chord diagram showing the recombination events in CoNS genomes from different animal hosts regardless of CoNS species. For visual clarity, only animal hosts with the highest number of isolates sampled are include in which recombination was detected. Recombination shown here includes recombination events detected in all CoNS species from the three animal hosts. For both panels, each line in the center connects a pair of donor and recipient genomes. For each line, the recipient genome is indicated by the end of the line ending nearer the outside of the plot and the potential donor cluster is indicated by the end of the line nearer the center of the plot. For inter-species recombination, the color of the connecting line is based on the color of the recipient genome.

We next sought to identify donor-recipient pairs of recombination events to determine if there were pairs that were more frequently exchanging DNA than with others. We identified 1659 and 223 recombination events involving genomes of the same species and between species, respectively (Fig. 6a and Supplementary Data 7). We detected the presence of intra-species recombination in 13 of the 15 CoNS species. The lack of intra-species recombination detected in *S. coagulans* and *S. succinus* is likely due to the low number of genomes in these species. *S. simulans* had the highest number of recombination events (*n* = 719), although this may be due to more frequent samples from this species. Nonetheless, these recombination events were detected in 3867 genes, indicating that 18.59% of the species pan-genome has been affected by recombination originating from other members of its own species. Other species that have high number of intra-species recombination events included *S. felis* (*n* = 202 recombination events in 163 genes), *S. xylosus* (*n* = 195 recombination events in 164 genes), *S. epidermidis* (*n* = 179 recombination events in 139 genes), *S. hominis* (*n* = 160 recombination events in 138 genes), and *S. chromogenes* (*n* = 69 recombination events in 62 genes). The most frequently recombining genes between isolates of the same species and that have known functions were the surface protein *pls* (21 recombination events in *S. simulans*) and iron-regulated surface determinant protein B *isdA* (9 events in *S. simulans*). Recombination was also detected in hypothetical proteins, with 12 and 10 recombination events in *S. epidermidis* and *S. felis*, respectively.

We also identified donor-recipient partners in inter-species recombination events, although these were not as frequent as intra-species recombination (Fig. 6a). All 15 CoNS species were involved in inter-species recombination. *S. simulans* was the most frequent DNA donor and DNA recipient, although this is likely due to this species having the highest number of genomes in our dataset. Regardless, we identified a total of 88 pairs of two CoNS species which are linked by at least one recombination event between them. The most frequent recombination partners were the sister taxa *S. pseudoxylosus* and *S. xylosus*, which were both frequent donors and recipients of each other. We identified 42 recombination events involving *S. pseudoxylosus* donors and *S. xylosus* recipients, whereas 11 recombination events involving *S. xylosus* donors and *S. pseudoxylosus* recipients. We also detected eight recombination events between *S. epidermidis* (donor) and *S. hominis* (recipient). Between species, the most frequently shared genes involved four recombination events involving the catalase gene [*S. xylosus* (donor) and *S. delphini* (recipient)] and site-specific integrase gene [*S. warneri* (donor) and *S. epidermidis* (recipient)] coding sequences, whereas there were three recombination events for the sharing of arsenite efflux transport membrane protein *arsB* between *S. epidermidis* (donor) and *S. hominis* (recipient). Overall, these results revealed that certain donor and recipient lineages exchange DNA more often with each other than they do with other lineages.

To further investigate the frequency of recombination of CoNS from within the same animal host species or between host species, we mapped all recombination events in any CoNS genome from the three most common animal groups sampled (Fig. 6b). These included 69 genomes from felines, 42 genomes from canines, and 27 genomes from bovines, representing a total of 72.25% (*n* = 138 genomes) of our CoNS dataset. We identified a total of 587 recombination events that occurred in 431 genes. The number of recombination events between isolates regardless of CoNS species that were from the same animal host were 190, 91, and 59 for feline, bovine, and canine hosts, respectively. The number of recombination events between isolates regardless of CoNS species that were from different animal hosts were: 63 between canines (donor) and felines (recipient), 59 between felines (donor) and canines (recipient), 41 between canines (donor) and bovine (recipient), 33 between bovine (donor) and canines (recipient), 29 between bovine (donor) and felines (recipient), and 22 between felines (donor) and bovine (recipient). These results show that recombination between CoNS genomes is frequent regardless of the identity of the animal host.

## Discussion

We present a comparative population analysis of 191 CoNS genomes sampled from diseased animals diagnosed in a veterinary diagnostic laboratory located in the northeast region of the United States. Our findings suggest that genetic exchange within and between species, though frequent, is not random nor indiscriminate. First, while often misclassified or overlooked in sampling efforts^1^, CoNS are important reservoirs of diverse phages, plasmids and mobilizable genes encoding AMR, heavy metal resistance, and virulence. The most frequently exchanged genes through homologous recombination were those associated with surface proteins, which may facilitate common strategies of different CoNS species to adapt to the same animal hosts. Second, we identified frequent exchange of DNA between certain donor-recipient partners, suggesting a structure to the genetic flux in which specific lineages act as hubs of gene sharing. Third, we detected frequent recombination between CoNS strains and species regardless of their animal host species, indicating that ecological barriers to recombination^32^ can be surmounted in co-circulating lineages. We interpret these findings as implying that frequent but structured patterns of recombination exist within and between CoNS species, which are driven by their overlapping ecological niches and geographical proximity.

The consequences of horizontal gene transfer followed by homologous recombination are vast. It is known to influence a myriad of evolutionary and population processes, including levels of standing diversity, niche expansion, spread of resistance and virulence determinants, and rapid adaptive changes in response to new or fluctuating environmental conditions^33,34^. Horizontal gene transfer can also result to the emergence of novel genetic variants or hybrids with unique phenotypes such as multidrug resistance, hyper-virulence, and altered transmissibility^35,36^. However, current models of gene transfer via recombination incorporate the null expectation that recombination is a homogeneous process across the species^37,38^. Such models lead to classifying bacteria as either clonal and panmictic species. In clonal species (e.g., *Mycobacterium tuberculosis*^39^), genetic variation between strains is generated mainly by mutation and emerging under neutral evolution through the random birth and extinction of lineages. In a panmictic species (e.g., *Helicobacter pylori*^40^), rates of recombination approach those found in sexual eukaryotes. High rates of recombination can theoretically prevent the separation of distinct daughter lineages, creating indistinct or fuzzy boundaries between them^41,42^. Although many studies have generated crucial insights into the nature and frequencies of recombination between bacterial species^34,37,38^, it is often assumed that all strains recombine at a uniform frequency and randomly across the entire species.

Our findings revealed that frequencies of recombination can vary dramatically even between strains of the same species, with a unique pattern of exchange for different strains and lineages. This pattern has been also observed in major bacterial pathogenic species^43–45^. Some strains donate or receive DNA more often than others^46,47^, while some strains tend to preferentially recombine with specific partners^44,48^. In the current study, we show that such variable patterns of recombination exist not only within species but also between species. The role of the animal host is particularly important in shaping patterns and frequencies of inter-species recombination in CoNS, as has also been demonstrated in the genus *Campylobacter*^32^. Differences in recombination frequencies also suggest that lineages respond to selective pressures in different ways. Such variation also implies that recombination itself can evolve in response to natural selection^49,50^ and can occur quickly on an evolutionary timescale^51,52^.

Bacterial strains that contain large tracts of recombined DNA^53^ are particularly intriguing because unpredictable phenotypes with considerable public health threats may arise. These strains reflect a mode of evolution that proceeds in jumps (or saltation) brought about by sudden and large genomic modifications and that have profound phenotypic consequences^54–56^. Frequently recombining strains may be likened to bacterial “hopeful monsters”, whereby most cases of rapid and dramatic changes to the genome are likely to be deleterious, but may occasionally be highly adaptive^54,55^. Such strains that contain large segments of recombined DNA have been reported in *Streptococcus pneumoniae*^44^, *Klebsiella pneumoniae*^57^, and *Vibrio anguillarum*^58^. The five genomes in our study that contain large tracts of recombined DNA as well as a few highly recombining genomes in *S. epidermidis* and *S. felis* seem to emerge as “hopeful monsters.” The origins of these hyper-recombinants and the factors that facilitate their emergence remain unclear, but close phylogenetic relationships, similar ecological niches, and geographical proximity can amplify opportunities for frequent and large-scale recombination events to occur. Investigations on the long-term evolutionary trajectory and phenotypic characteristics of hyper-recombining CoNS will have profound implications to effectively managing CoNS infections.

Our results should be interpreted within the confines of the study limitations. First, we acknowledge limitations associated with sampling bias. Each CoNS species was disproportionately represented by variable number of genomes. Our dataset is also heavily biased to favor some animal groups more than others, with more isolates recovered from feline, canine, and bovine hosts. These two sources of bias paint an uneven characterization of genome content per species, including the frequencies and patterns of recombination within and among CoNS species and genomes. Future research should also spotlight CoNS-specific genetic elements, especially those that may contribute to their ecological adaptation, drug resistance, and pathogenesis. A broader and systematic sampling strategy will be instrumental in uncovering novel CoNS species and lineages from an even more diverse range of animal species and their mechanisms of DNA mobility. This is especially true at the human-animal interface where pathogen transmission is most likely to occur. It will also help in establishing the host range of each CoNS species and in identifying those species that are restricted to a single animal species.

In summary, our results underscore the impact of frequent but skewed patterns of homologous recombination, mobile genetic elements, and accessory gene sharing in shaping the diversity, genome dynamics, and host adaptation of CoNS. These data will be important in developing effective surveillance, diagnostic, and treatment options for CoNS infections.

## Methods

### Bacterial sampling

The CoNS culture collection consisted of 191 isolates that were retrospectively sampled from September 2017 through March 2020. A total of 18, 67, 87, and 19 isolates were collected in 2017, 2018, 2019, and 2020, respectively. Isolates were obtained as culture swabs from routine clinical specimens submitted to the NHVDL, New Hampshire, USA. Isolates were mostly recovered from body sites such as ear (*n* = 35), animal milk (*n* = 26), skin (*n* = 24), wounds (*n* = 17), and urine (*n* = 16). The clinical specimens were received from multiple veterinary practices from the New England region of the country, consisting of the states of Connecticut, New Hampshire, Maine, Massachusetts, and Vermont. We also included two isolates where the animals were from Alabama (located in the southern part of the country) but were diagnosed in New England. These were included in our analysis because it was unknown whether they were infected while they were in New England. All isolates were from animals with confirmed clinical infections. No live vertebrates were used in this study; hence, the NHVDL was exempt from the International Animal Care and Use Committee (IACUC) approval process. Pure isolates were cultured in commercially prepared tryptic soy agar with 10% sheep red blood cells and brain heart infusion broth. Preliminary identification of CoNS species was carried out using a Brucker Biotyper MALDI-TOF MS in accordance to the manufacturer’s instructions. The machine determines proteomic fingerprint of the organism under query and matches it against two libraries of reference spectra RUO library 6903(V6) and 7311(V7) available in the Bruker MBT Compass (Bruker Daltonics, Bremen, Germany). All isolates were stored in DMSO solution in −80 °C. Associated metadata information for each isolate, including date of sample collection, location, and isolation source, are included in Supplementary Data 1.

### Methicillin susceptibility screening

In vitro screening for cefoxitin and oxacillin resistance was carried out using the Kirby Bauer disc diffusion technique. Because there are no breakpoint guidelines in the most current Clinical and Laboratory Standards Institute (CLSI) for CoNS species, we used the CLSI breakpoint guidelines for cefoxitin which is used as the official predictor of methicillin resistance for *S. aureus*^59^. For isolates identified as methicillin resistant, we determined the presence of the penicillin-binding protein PBP2 using a commercial latex agglutination test (MASTALEX MRSA Latex Kit, MAST, UK) following manufacturer’s guidelines. We confirmed the presence of the *mecA* gene by screening the genome sequence of each isolate (described below).

### DNA extraction and whole genome sequencing

Isolates were grown overnight in brain heart infusion broth at 37 °C. Genomic DNA from overnight cultures were then extracted using the Zymo Quick DNA Fungal/Bacterial Miniprep Kit (Irvine, California) following manufacturer’s instructions. DNA quantity was measured using a Qubit fluorometer (Invitrogen, Grand Island, NY) according to manufacturer’s instructions. Genome sequencing was carried out using the NextSeq 2000 platform at the Microbial Genome Sequencing Center (now SeqCenter) in Pittsburgh, Pennsylvania in 2022. Sample libraries were prepared using the Illumina DNA Preparation kit and IDT 10 bp UDI indices following the manufacturer’s instructions. Sequencing generated paired-end reads (2 × 151 bp) on multiplexed libraries. Demultiplexing, quality control, and adapter trimming were performed with the Illumina bcl-convert (v3.9.3).

### Genome assembly, quality check, annotation, and species designation

We used the shovill v.1.1.0 pipeline to assemble all paired-end reads (https://github.com/tseemann/shovill). Shovill uses the SPAdes assembly algorithm^60^ but alters several pre- and post-assembly steps to generate similar and high-quality assembly results in less time. We used the --trim option to enable adapter trimming and improve assembly. To assess quality of the assembled genomes, we used QUAST v.5.0.2^61^. We excluded assemblies with >200 contigs and an N50 < 40,000 bp. We used CheckM v.1.1.3 to determine only those genomes with level of completeness of >90% and contamination of <5%^62^. We calculated the genome completeness (mean = 99.31%; range: 97.20–99.81%) and genome contamination (mean = 0.9%; range = 0 to 4.44%), which were all within the genome quality standards recommended by CheckM (Supplementary Data 1 and Fig. 1). To determine genomic relatedness and delineate species boundaries, we calculated the genome-wide Average Nucleotide Identity (ANI) for every possible pairs of genomes using fastANI v.1.32^21^. ANI refers to the mean nucleotide identity of orthologous pair of genes that are shared between a pair or collection of microbial genomes^21^. We used the >95% ANI threshold to confirm species identification. The draft genomes were annotated using Prokka v.1.14.6^63^ and polished with Bakta^64^.

### Pan-genome analysis and phylogenetic tree reconstruction

To characterize the pan-genome, we used PIRATE v.1.0.4, a fast and scalable platform for clustering orthologous gene families in bacteria^65^. Briefly, gene orthologs are clustered over the default identity threshold values ranging from 50% to 98% (50, 60, 70, 90, 90, 95, 98) sequence identity using CD-HIT^66^. A gene presence and absence matrix were produced in tab separated file using the PIRATE supplement Rtab.pl script. Gene sequences were aligned using MAFFT^67^. Sequence alignments of core genes (i.e., gene families present in >95% of genomes) were concatenated to generate the core genome alignment. Single nucleotide polymorphisms (SNPs) were extracted from the core genome alignment using SNP-sites^68^. The core SNP alignment was used as input for building a maximum likelihood phylogenetic tree using RAxML v.8.2.12^69^. We used the general time reversible model for nucleotide substitution^70^ under the GAMMA model of rate heterogeneity. Phylogenetic trees were visualized and annotated using figtree v.1.4.4 (http://tree.bio.ed.ac.uk/software/figtree/) and Interactive Tree of Life IToL^71^.

### Accessory gene network analysis

The gene presence or absence matrix generated using PIRATE, excluding the core genes, were used as input in GraPPLE (https://github.com/JDHarlingLee/GraPPLE). Briefly, a Jaccard similarity coefficient based on the number of shared genes over the total number of genes across a pair of genomes was calculated using the pw_similarity.py script. Metadata were added using the metadata_to_layout.py script. Clustering by accessory gene content similarity was visualized as networks using Graphia^72^. Accessory genome similarity was clustered using MCL - Markov algorithm. Edges of networks were transformed using the k-nearest neighbor algorithm (k = 5).

### In silico detection of antimicrobial resistance genes, virulence genes, SCC*mec*, plasmid replicons, and phages

Genome assemblies were screened for the presence of acquired antimicrobial resistance genes and heavy metal resistance genes using the National Centre for Biotechnology Information’s (NCBI) AMRFinderPlus v.3.10.23 and its accompanying NCBI-compiled AMR database^73^. We also screened the genome assemblies for the presence of virulence determinants using ABRicate v.1.0.1 (https://github.com/tseemann/abricate) containing the Virulence Factor Database (VFDB)^74^. We also used ABRicate to search for the *rep* gene that codes for the plasmid replicon initiator protein (*rep*) against the Plasmid Finder database^30^. We used staphopia-sccmec^75^ to carry out in silico detection and classification of SCCmec. Staphophia-sccmec uses a primer-based approach where assemblies are aligned against SCC*mec* typing primers^75^. Samples with a perfect match are assigned an SCC*mec* type. We used VirSorter2 (https://github.com/jiarong/VirSorter2)^76^ to determine phage diversity in CoNS genomes. For each genome, the length of phage DNA regions was summed to give total length in each genome.

### Inference of homologous recombination

Using the sequence alignments of individual core genes and shared accessory genes, we inferred recent and ancestral recombination events used fastGEAR^31^ (Supplementary Data 9). FastGEAR identifies lineages in the alignments and implements a Hidden Markov Model to compare polymorphic sites occurring in individual strain and compare them to other polymorphic sites occurring in members of its own lineage as well as strains from other lineages. The output of fastGEAR is parsed into HERO (Highways Enumerated by Recombination Events) (https://github.com/therealcooperpark/hero), a pipeline implemented in Python to visualize donor-recipient strain pairs in recent recombination events identified by fastGEAR. Visualization of recombination events was carried out using Circos v.0.69-8^77^.

### Statistics and reproducibility

To test the significance of the inferred recombinations and identify false-positive results, we used a diversity test implemented in fastGEAR^31^. This is based on a simple binomial test which computes a Bayes factor (=1) that measures how different the SNP density changed between the DNA fragment in question compared to its background (lineage). We calculated the Pearson correlation coefficient implemented in R package ggpubr v0.4.0 to determine the association between core genome SNPs and ANI of every genome per species. We used a *p*-value threshold of <0.001.

### Reporting summary

Further information on research design is available in the Nature Portfolio Reporting Summary linked to this article.

## Supplementary information

Supplemental Information

Description of Additional Supplementary Files

Supplemental Data 1-9

Reporting Summary

## Data Availability

The dataset supporting the conclusions of this article is included within the article and its supplementary files. Genome sequence data of CoNS isolates have been deposited in the NCBI Sequence Read Archive under BioProject accession number PRJNA870509. BioSample accession numbers for each genome are listed in Supplementary Data 1.

## References

[1] Becker K, Heilmann C, Peters G (2014). Coagulase-negative staphylococci. Clin. Microbiol. Rev..

[2] Otto M (2010). Staphylococcus colonization of the skin and antimicrobial peptides. Expert Rev. Dermatol..

[3] Berends MS (2022). Trends in occurrence and phenotypic resistance of coagulase-negative Staphylococci (CoNS) found in human blood in the northern Netherlands between 2013 and 2019. Microorganisms.

[4] Michalik M (2020). Coagulase-negative staphylococci (CoNS) as a significant etiological factor of laryngological infections: a review. Ann. Clin. Microbiol. Antimicrob..

[5] Akinduti PA (2022). Emerging vancomycin-non susceptible coagulase negative Staphylococci associated with skin and soft tissue infections. Ann. Clin. Microbiol. Antimicrob..

[6] Azimi T (2020). Coagulase-negative staphylococci (CoNS) meningitis: a narrative review of the literature from 2000 to 2020. N. Microbes N. Infect..

[7] Tornero E (2012). Prosthetic joint infections due to *Staphylococcus aureus* and coagulase-negative staphylococci. Int. J. Artif. Organs.

[8] Hebeisen UP, Atkinson A, Marschall J, Buetti N (2019). Catheter-related bloodstream infections with coagulase-negative staphylococci: are antibiotics necessary if the catheter is removed?. Antimicrob. Resist Infect. Control.

[9] Chu VH (2008). Emergence of coagulase-negative staphylococci as a cause of native valve endocarditis. Clin. Infect. Dis..

[10] May L, Klein EY, Rothman RE, Laxminarayan R (2014). Trends in antibiotic resistance in coagulase-negative staphylococci in the United States, 1999 to 2012. Antimicrob. Agents Chemother..

[11] Pedroso SHSP (2018). Coagulase-negative staphylococci isolated from human bloodstream infections showed multidrug resistance profile. Micro. Drug Resist.

[12] Michels R, Last K, Becker SL, Papan C (2021). Update on coagulase-negative Staphylococci-what the clinician should know. Microorganisms.

[13] Everitt RG (2014). Mobile elements drive recombination hotspots in the core genome of *Staphylococcus aureus*. Nat. Commun..

[14] Driebe EM (2015). Using whole genome analysis to examine recombination across diverse sequence types of *Staphylococcus aureus*. PLoS ONE.

[15] Murray S (2017). Recombination-mediated host adaptation by avian *Staphylococcus aureus*. Genome Biol. Evol..

[16] Bouchami O, de Lencastre H, Miragaia M (2016). Impact of insertion sequences and recombination on the population structure of Staphylococcus haemolyticus. PLoS One.

[17] Datta MS (2021). Rapid methicillin resistance diversification in *Staphylococcus epidermidis* colonizing human neonates. Nat. Commun..

[18] Smith JT, Andam CP (2021). Extensive horizontal gene transfer within and between species of coagulase-negative Staphylococcus. Genome Biol. Evol..

[19] Didelot X, Maiden MCJ (2010). Impact of recombination on bacterial evolution. Trends Microbiol..

[20] Choi SC (2012). Replacing and additive horizontal gene transfer in Streptococcus. Mol. Biol. Evol..

[21] Jain C, Rodriguez-R LM, Phillippy AM, Konstantinidis KT, Aluru S (2018). High throughput ANI analysis of 90K prokaryotic genomes reveals clear species boundaries. Nat. Commun..

[22] Hartman BJ, Tomasz A (1984). Low-affinity penicillin-binding protein associated with beta-lactam resistance in *Staphylococcus aureus*. J. Bacteriol..

[23] International Working Group on the Classification of Staphylococcal Cassette Chromosome Elements (IWG-SCC (2009). Classification of staphylococcal cassette chromosome mec (SCCmec): guidelines for reporting novel SCCmec elements. Antimicrob. Agents Chemother..

[24] García-Álvarez L (2011). Meticillin-resistant *Staphylococcus aureus* with a novel mecA homologue in human and bovine populations in the UK and Denmark: a descriptive study. Lancet Infect. Dis..

[25] Diemond-Hernández B, Solórzano-Santos F, Leaños-Miranda B, Peregrino-Bejarano L, Miranda-Novales G (2010). Production of icaADBC-encoded polysaccharide intercellular adhesin and therapeutic failure in pediatric patients with Staphylococcal device-related infections. BMC Infect. Dis..

[26] Sundaramoorthy R, Fyfe PK, Hunter WN (2008). Structure of *Staphylococcus aureus* EsxA suggests a contribution to virulence by action as a transport chaperone and/or adaptor protein. J. Mol. Biol..

[27] Cheung GYC (2015). Functional characteristics of the *Staphylococcus aureus* δ-toxin allelic variant G10S. Sci. Rep..

[28] Frost LS, Leplae R, Summers AO, Toussaint A (2005). Mobile genetic elements: the agents of open source evolution. Nat. Rev. Microbiol..

[29] Oliveira H (2019). Staphylococci phages display vast genomic diversity and evolutionary relationships. BMC Genom..

[30] Carattoli A, Hasman H (2020). PlasmidFinder and in silico pMLST: identification and typing of plasmid replicons in whole-genome sequencing (WGS). Methods Mol. Biol..

[31] Mostowy R (2017). Efficient inference of recent and ancestral recombination within bacterial populations. Mol. Biol. Evol..

[32] Mourkas E (2022). Host ecology regulates interspecies recombination in bacteria of the genus Campylobacter. Elife.

[33] Hanage WP (2016). Not so simple after all: bacteria, their population genetics, and recombination. Cold Spring Harb. Perspect. Biol..

[34] Levin BR, Cornejo OE (2009). The population and evolutionary dynamics of homologous gene recombination in bacterial populations. PLoS Genet..

[35] Perron GG, Lee AEG, Wang Y, Huang WE, Barraclough TG (2012). Bacterial recombination promotes the evolution of multi-drug-resistance in functionally diverse populations. Proc. Biol. Sci..

[36] Spoor LE (2015). Recombination-mediated remodelling of host-pathogen interactions during *Staphylococcus aureus* niche adaptation. Micro. Genom..

[37] Vos M, Didelot X (2009). A comparison of homologous recombination rates in bacteria and archaea. ISME J..

[38] González-Torres P, Rodríguez-Mateos F, Antón J, Gabaldón T (2019). Impact of homologous recombination on the evolution of prokaryotic core genomes. MBio.

[39] Dos Vultos T (2008). Evolution and diversity of clonal bacteria: the paradigm of *Mycobacterium tuberculosis*. PLoS ONE.

[40] Yahara K (2012). Genome-wide survey of mutual homologous recombination in a highly sexual bacterial species. Genome Biol. Evol..

[41] Hanage WP, Fraser C, Spratt BG (2005). Fuzzy species among recombinogenic bacteria. BMC Biol..

[42] Corander J, Connor TR, O’Dwyer CA, Kroll JS, Hanage WP (2012). Population structure in the Neisseria, and the biological significance of fuzzy species. J. R. Soc. Interface.

[43] Mostowy R (2014). Heterogeneity in the frequency and characteristics of homologous recombination in pneumococcal evolution. PLoS Genet..

[44] Chewapreecha C (2014). Dense genomic sampling identifies highways of pneumococcal recombination. Nat. Genet..

[45] Sakoparnig T, Field C, van Nimwegen E (2021). Whole genome phylogenies reflect the distributions of recombination rates for many bacterial species. Elife.

[46] Rodríguez-Beltrán J (2015). High recombinant frequency in extraintestinal pathogenic Escherichia coli strains. Mol. Biol. Evol..

[47] Wyres KL (2019). Distinct evolutionary dynamics of horizontal gene transfer in drug resistant and virulent clones of Klebsiella pneumoniae. PLoS Genet..

[48] Park CJ, Andam CP (2020). Distinct but intertwined evolutionary histories of multiple Salmonella enterica subspecies. mSystems.

[49] Lobkovsky AE, Wolf YI, Koonin EV (2015). Evolvability of an optimal recombination rate. Genome Biol. Evol..

[50] Peñalba JV, Wolf JBW (2020). From molecules to populations: appreciating and estimating recombination rate variation. Nat. Rev. Genet..

[51] Evans BA, Rozen DE (2013). Significant variation in transformation frequency in Streptococcus pneumoniae. ISME J..

[52] Cowley LA (2018). Evolution via recombination: cell-to-cell contact facilitates larger recombination events in Streptococcus pneumoniae. PLoS Genet..

[53] Hanage WP, Fraser C, Tang J, Connor TR, Corander J (2009). Hyper-recombination, diversity, and antibiotic resistance in pneumococcus. Science.

[54] Goldschmidt R (1933). Some aspects of evolution. Science.

[55] Theissen G (2009). Saltational evolution: hopeful monsters are here to stay. Theory Biosci..

[56] Katsnelson MI, Wolf YI, Koonin EV (2019). On the feasibility of saltational evolution. Proc. Natl Acad. Sci. USA.

[57] Chen L, Mathema B, Pitout JDD, DeLeo FR, Kreiswirth BN (2014). Epidemic Klebsiella pneumoniae ST258 is a hybrid strain. mBio.

[58] Coyle NM (2020). A hopeful sea-monster: a very large homologous recombination event impacting the core genome of the marine pathogen Vibrio anguillarum. Front. Microbiol..

[59] Clinical and Laboratory Standards Institute (CLSI). *CLSI Performance Standards for Antimicrobial Disk and Dilution Susceptibility Tests for Bacteria Isolated from Animals. VET01S*. (2021).

[60] Bankevich A (2012). SPAdes: a new genome assembly algorithm and its applications to single-cell sequencing. J. Comput Biol..

[61] Gurevich A, Saveliev V, Vyahhi N, Tesler G (2013). QUAST: quality assessment tool for genome assemblies. Bioinformatics.

[62] Parks DH, Imelfort M, Skennerton CT, Hugenholtz P, Tyson GW (2015). CheckM: assessing the quality of microbial genomes recovered from isolates, single cells, and metagenomes. Genome Res..

[63] Seemann T (2014). Prokka: rapid prokaryotic genome annotation. Bioinformatics.

[64] Schwengers O (2021). Bakta: rapid and standardized annotation of bacterial genomes via alignment-free sequence identification. Micro. Genom..

[65] Bayliss SC, Thorpe HA, Coyle NM, Sheppard SK, Feil EJ (2019). PIRATE: a fast and scalable pangenomics toolbox for clustering diverged orthologues in bacteria. Gigascience.

[66] Fu L, Niu B, Zhu Z, Wu S, Li W (2012). CD-HIT: accelerated for clustering the next-generation sequencing data. Bioinformatics.

[67] Katoh K, Asimenos G, Toh H (2009). Multiple alignment of DNA sequences with MAFFT. Methods Mol. Biol..

[68] Page AJ (2016). SNP-sites: rapid efficient extraction of SNPs from multi-FASTA alignments. Micro. Genom..

[69] Stamatakis A (2014). RAxML version 8: a tool for phylogenetic analysis and post-analysis of large phylogenies. Bioinformatics.

[70] Tavaré S (1986). Some probabilistic and statistical problems in the analysis of DNA sequences. Am. Math. Soc.: Lect. Math. Life Sci..

[71] Letunic I, Bork P (2019). Interactive Tree Of Life (iTOL) v4: recent updates and new developments. Nucleic Acids Res..

[72] Freeman TC (2022). Graphia: a platform for the graph-based visualisation and analysis of high dimensional data. PLoS Comput. Biol..

[73] Feldgarden M (2021). AMRFinderPlus and the Reference Gene Catalog facilitate examination of the genomic links among antimicrobial resistance, stress response, and virulence. Sci. Rep..

[74] Liu B, Zheng D, Jin Q, Chen L, Yang J (2019). VFDB 2019: a comparative pathogenomic platform with an interactive web interface. Nucleic Acids Res..

[75] Petit RA, Read TD (2018). *Staphylococcus aureus* viewed from the perspective of 40,000+ genomes. PeerJ.

[76] Guo J (2021). VirSorter2: a multi-classifier, expert-guided approach to detect diverse DNA and RNA viruses. Microbiome.

[77] Krzywinski M (2009). Circos: an information aesthetic for comparative genomics. Genome Res..

