# Supplementary material for: Leaky barriers to gene sharing between locally co-existing coagulase-negative Staphylococcus species
Source: Commun Biol. 2023 May 3;6:482. doi: 10.1038/s42003-023-04877-0 (PMC10156822; doi:10.1038/s42003-023-04877-0)
Supplement: Supplementary file 1 — Supplemental Information [file 42003_2023_4877_MOESM1_ESM.pdf]

Leaky barriers to gene sharing between locally co-existing coagulase-negative Staphylococcus species

Odion O. Ikhimiukor, Stephanie S. R. Souza, Michael M. Marcovici, Griffin J. Nye, Robert Gibson, Cheryl P. Andam

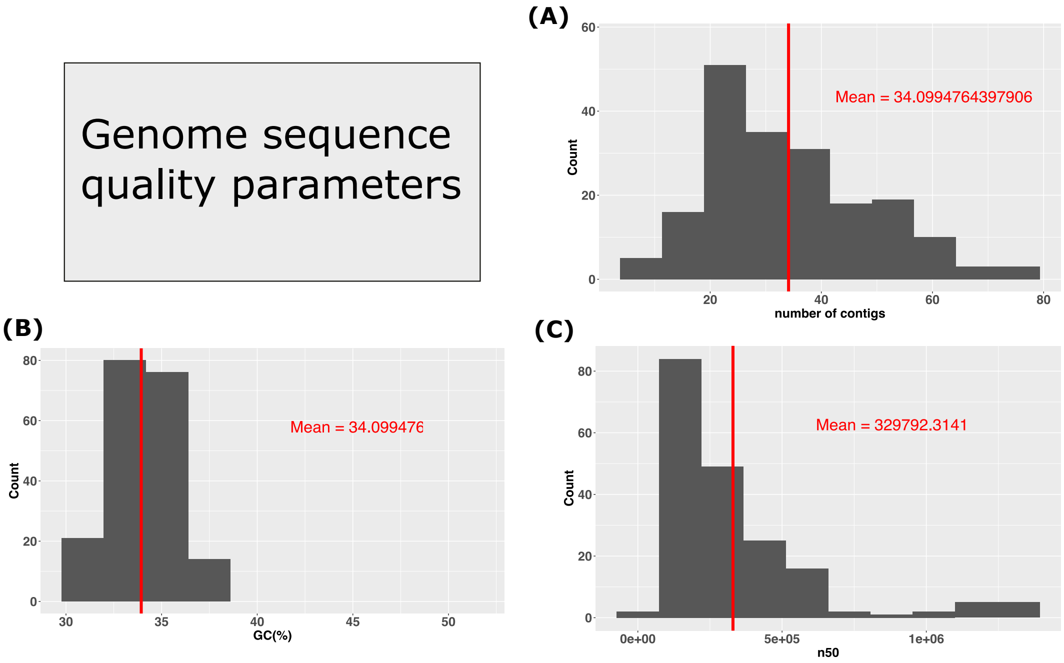

Supplementary Figure 1. Sequence quality metrics of the 191 CoNS genomes used in this study.

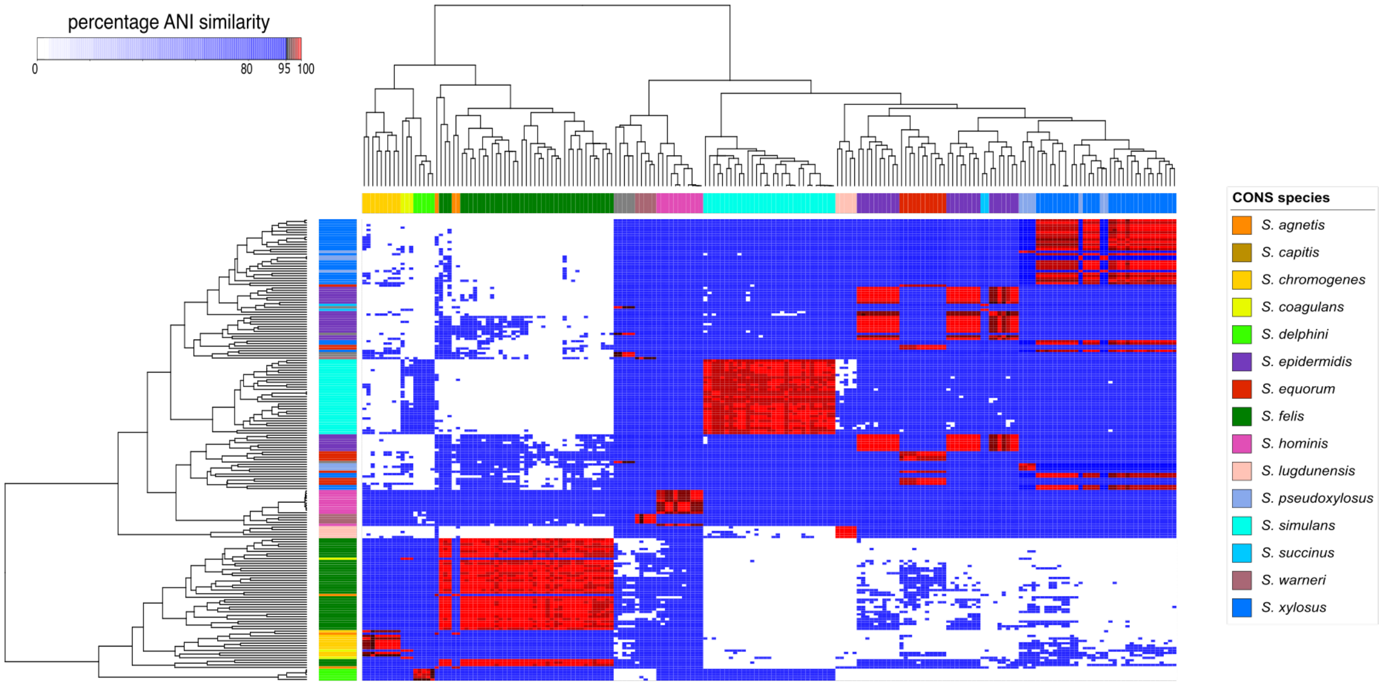

Supplementary Figure 2. Genome-wide average nucleotide identity (ANI) for all pairs of CoNS genomes. The 95% ANI similarity threshold (red) was used to delineate species boundaries.

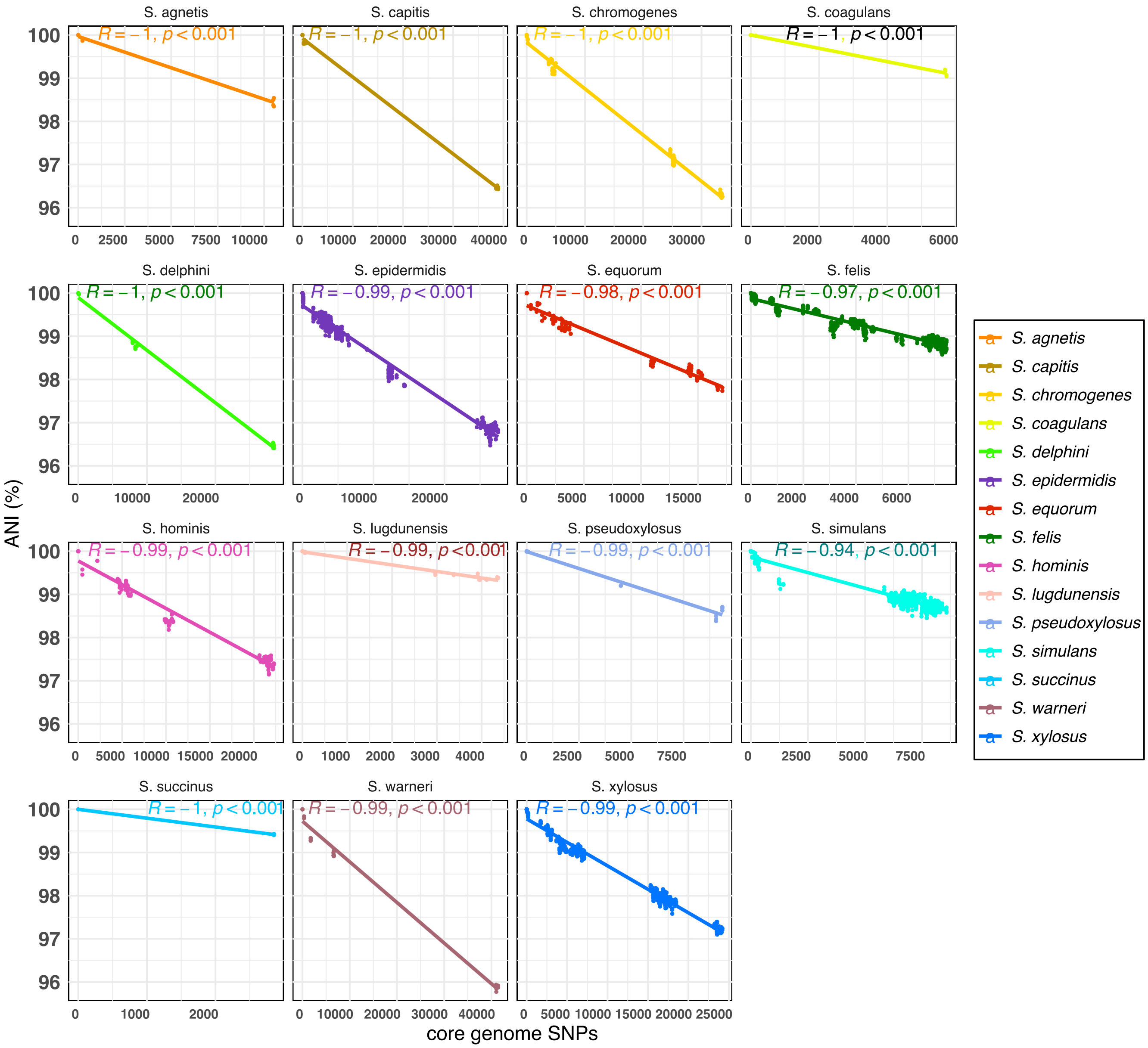

Supplementary Figure 3. Scatter plot of pairwise ANI similarities and core genome SNP distance between genome pairs for every CoNS species. Association between the two variables was tested using the Pearson correlation coefficient. Only the most common CoNS species are included.

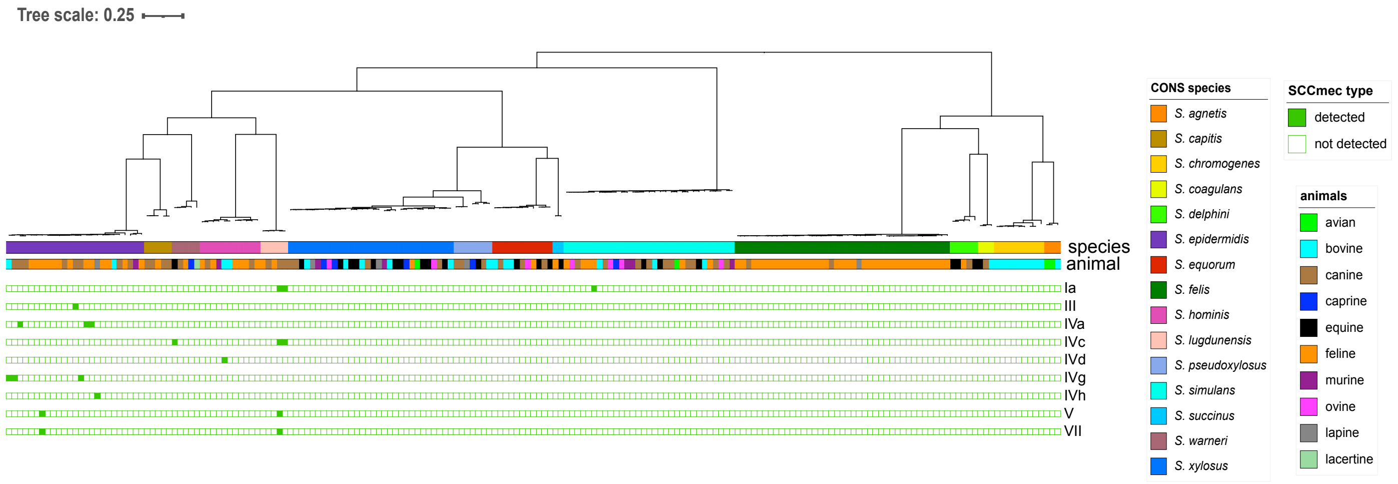

Supplementary Figure 4. Types and distribution of the *mecA*-carrying chromosomal cassette SCC*mec*. The tree is identical to that found in Figure 2a.

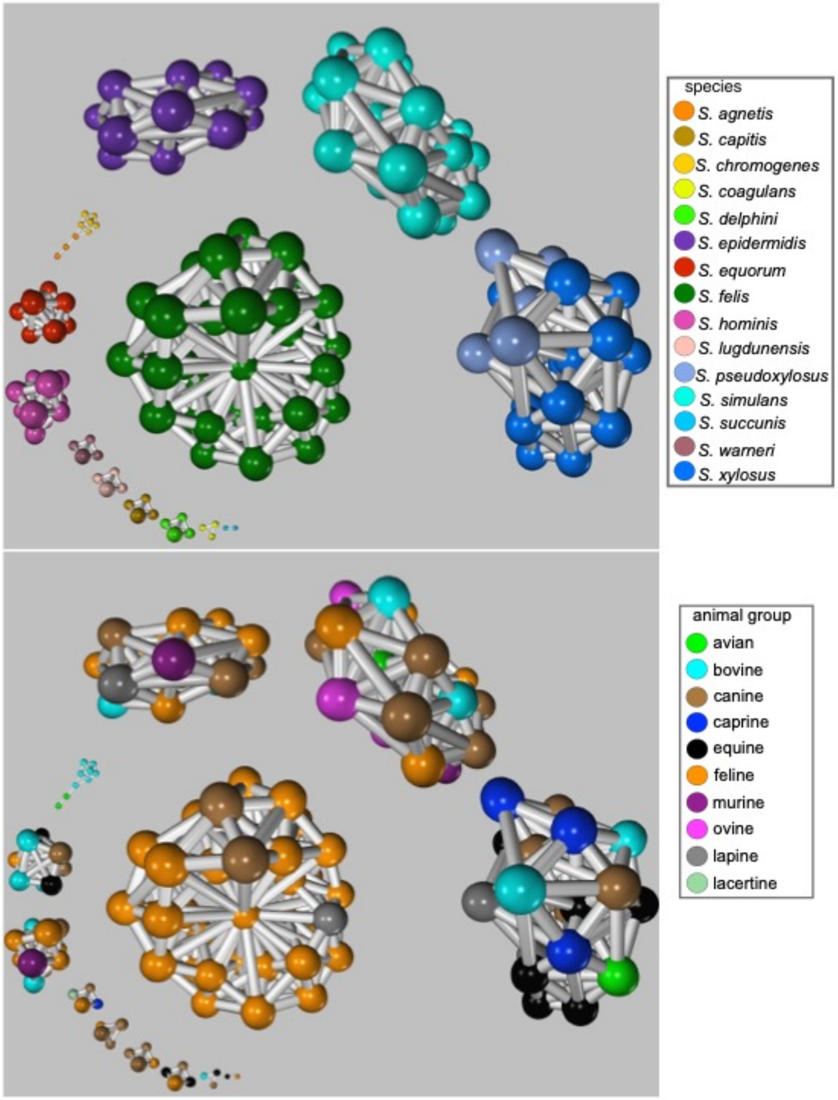

Supplementary Figure 5. Network analysis of accessory genomes of CoNS isolates. Edges refer to distances between the genomes in a cluster. Each node is colored by (a) species assignment per genome. (b) animal group of isolates.
