# Supplementary material for: Leaky barriers to gene sharing between locally co-existing coagulase-negative Staphylococcus species
Source: Commun Biol. 2023 May 3;6:482. doi: 10.1038/s42003-023-04877-0 (PMC10156822; doi:10.1038/s42003-023-04877-0)
Supplement: Supplementary file 2 — Description of Additional Supplementary Files [file 42003_2023_4877_MOESM2_ESM.pdf]

Description of Additional Supplementary Files

File name: Supplementary Data 1
Description: Metadata, sequence quality metrics, and NCBI Accession numbers for each CoNS isolate in this study

File name: Supplementary Data 2
Description: Genome-wide average nucleotide identity (ANI) for all pairs of CoNS genomes. The 95% ANI similarity threshold (red) was used to delineate species boundaries

File name: Supplementary Data 3
Description: Presence (yes) or absence (no) of antimicrobial resistance genes, heavy metal resistance genes, and virulence genes in each of the 191 CoNS genomes

File name: Supplementary Data 4
Description: Types and distribution of the mecA-carrying chromosomal cassette SCCmec. No means the SCCmec was not detected, while yes means the SCCmec was detected in the genome

File name: Supplementary Data 5
Description: The presence of phage DNA and associated genes per CoNS genome

File name: Supplementary Data 6
Description: Presence and types of the plasmid replicon initiator protein per CoNS genome

File name: Supplementary Data 7
Description: Total lengths of recombined DNA in each of the 191 CoNS genomes

File name: Supplementary Data 8
Description: Donors and recipients of recombination events inferred using fastGEAR

File name: Supplementary Data 9
Description: Recent and ancestral recombination events inferred using fastGEAR
