# Supplementary material for: Leaky barriers to gene sharing between locally co-existing coagulase-negative Staphylococcus species
Source: Commun Biol. 2023 May 3;6:482. doi: 10.1038/s42003-023-04877-0 (PMC10156822; doi:10.1038/s42003-023-04877-0)
Supplement: Supplementary file 4 — Reporting Summary [file 42003_2023_4877_MOESM4_ESM.pdf]

nature portfolio

Corresponding author(s): Andam

Last updated by author(s): Mar 23, 2023

Reporting Summary

Nature Portfolio wishes to improve the reproducibility of the work that we publish. This form provides structure for consistency and transparency in reporting. For further information on Nature Portfolio policies, see our Editorial Policies and the Editorial Policy Checklist.

Statistics

For all statistical analyses, confirm that the following items are present in the figure legend, table legend, main text, or Methods section.

n/a	Confirmed	
☐	☒	The exact sample size (*n*) for each experimental group/condition, given as a discrete number and unit of measurement
☐	☒	A statement on whether measurements were taken from distinct samples or whether the same sample was measured repeatedly
☐	☒	The statistical test(s) used AND whether they are one- or two-sided<br>*Only common tests should be described solely by name; describe more complex techniques in the Methods section.*
☐	☒	A description of all covariates tested
☐	☒	A description of any assumptions or corrections, such as tests of normality and adjustment for multiple comparisons
☐	☒	A full description of the statistical parameters including central tendency (e.g. means) or other basic estimates (e.g. regression coefficient) AND variation (e.g. standard deviation) or associated estimates of uncertainty (e.g. confidence intervals)
☐	☒	For null hypothesis testing, the test statistic (e.g. *F*, *t*, *r*) with confidence intervals, effect sizes, degrees of freedom and *P* value noted<br>*Give P values as exact values whenever suitable.*
☒	☐	For Bayesian analysis, information on the choice of priors and Markov chain Monte Carlo settings
☒	☐	For hierarchical and complex designs, identification of the appropriate level for tests and full reporting of outcomes
☒	☐	Estimates of effect sizes (e.g. Cohen's *d*, Pearson's *r*), indicating how they were calculated

Our web collection on statistics for biologists contains articles on many of the points above.

Software and code

Policy information about availability of computer code

Data collection

No software was used for data collection.

Data analysis

Genome assembly, quality check, annotation and species designation
We used the shovill v.1.1.0 pipeline to assemble all paired end reads (https://github.com/tseemann/shovill). Shovill uses the SPAdes assembly algorithm but alters several pre- and post-assembly steps to generate similar and high-quality assembly results in less time. We used the --trim option to enable adapter trimming and improve assembly. To assess quality of the assembled genomes, we used QUAST v.5.0.2. We excluded assemblies with > 200 contigs and an N50 < 40,000 bp. We used CheckM v.1.1.3 to determine only those genomes with level of completeness of > 90% and contamination of < 5%. We calculated the genome completeness (mean = 99.31%; range: 97.20 - 99.81%) and genome contamination (mean = 0.9%; range = 0 to 4.44%), which were all within the genome quality standards recommended by CheckM (Supplementary Table S1 and Figure S1). To determine genomic relatedness and delineate species boundaries, we calculated the genome-wide Average Nucleotide Identity (ANI) for every possible pairs of genomes using fastANI v.1.32 ANI refers to the mean nucleotide identity of orthologous pair of genes that are shared between a pair or collection of microbial genomes. We used the > 95% ANI threshold to confirm species identification. The draft genomes were annotated using Prokka v.1.14.6 and polished with Bakta.

Pan-genome analysis and phylogenetic tree reconstruction
To characterize the pan-genome, we used PIRATE v.1.0.4, a fast and scalable platform for clustering orthologous gene families in bacteria 65. Briefly, gene orthologs are clustered over the default identity threshold values ranging from 50% to 98% (50, 60, 70, 90, 90, 95, 98) sequence identity using CD-HIT. A gene presence and absence matrix were produced in tab separated file using the PIRATE supplement Rtab.pl script. Gene sequences were aligned using MAFFT. Sequence alignments of core genes (i.e., gene families present in >95% of genomes) were concatenated to generate the core genome alignment. Single nucleotide polymorphisms (SNPs) were extracted from the core genome alignment using SNP-sites. The core SNP alignment was used as input for building a maximum likelihood phylogenetic tree using RAxML

v.8.2.12. We used the general time reversible model for nucleotide substitution under the GAMMA model of rate heterogeneity. Phylogenetic trees were visualized and annotated using figtree v.1.4.4 (http://tree.bio.ed.ac.uk/software/figtree/) and Interactive Tree of Life IToL.

Accessory gene network analysis
The gene presence or absence matrix generated using PIRATE, excluding the core genes, were used as input in GraPPLE (https://github.com/JDHarlingLee/GraPPLE). Briefly, a Jaccard similarity coefficient based on the number of shared genes over the total number of genes across a pair of genomes was calculated using the pw_similarity.py script. Metadata were added using the metadata_to_layout.py script. Clustering by accessory gene content similarity was visualized as networks using Graphia. Accessory genome similarity was clustered using MCL - Markov algorithm. Edges of networks were transformed using the k-nearest neighbour algorithm (k = 5).

In silico detection of antimicrobial resistance genes, virulence genes, SCCmec, plasmid replicons, and phages
Genome assemblies were screened for the presence of acquired antimicrobial resistance genes and heavy metal resistance genes using the National Centre for Biotechnology Information's (NCBI) AMRFinderPlus v.3.10.23 and its accompanying NCBI-compiled AMR database. We also screened the genome assemblies for the presence of virulence determinants using ABRicate v.1.0.1 (https://github.com/tseemann/abricate) containing the Virulence Factor Database (VFDB). We also used ABRicate to search for the rep gene that codes for the plasmid replicon initiator protein (rep) against the Plasmid Finder database. We used staphopia-sccmec to carry out in silico detection and classification of SCCmec. Staphophia-sccmec uses a primer-based approach where assemblies are aligned against SCCmec typing primers. Samples with a perfect match are assigned an SCCmec type. We used VirSorter2 (https://github.com/jiarong/VirSorter2) to determine phage diversity in CoNS genomes. For each genome, the length of phage DNA regions was summed to give total length in each genome.

Inference of homologous recombination
Using the sequence alignments of individual core genes and shared accessory genes, we inferred recent and ancestral recombination events used fastGEAR . FastGEAR identifies lineages in the alignments and implements a Hidden Markov Model to compare polymorphic sites occurring in individual strain and compare them to other polymorphic sites occurring in members of its own lineage as well as strains from other lineages. The output of fastGEAR is parsed into HERO (Highways Enumerated by Recombination Events) (https://github.com/therealcooperpark/hero), a pipeline implemented in Python to visualize donor-recipient strain pairs in recent recombination events identified by fastGEAR. Visualization of recombination events was carried out using Circos v.0.69-8.

Statistics and Reproducibility
To test the significance of the inferred recombinations and identify false-positive results, we used a diversity test implemented in fastGEAR 31. This is based on a simple binomial test which computes a Bayes factor (= 1) that measures how different the SNP density changed between the DNA fragment in question compared to its background (lineage). We calculated the Pearson correlation coefficient implemented in R package ggpubr v0.4.0 to determine the association between core genome SNPs and ANI of every genome per species. We used a p-value threshold of < 0.001.

For manuscripts utilizing custom algorithms or software that are central to the research but not yet described in published literature, software must be made available to editors and reviewers. We strongly encourage code deposition in a community repository (e.g. GitHub). See the Nature Portfolio guidelines for submitting code & software for further information.

Data

Policy information about availability of data

All manuscripts must include a data availability statement. This statement should provide the following information, where applicable:

- Accession codes, unique identifiers, or web links for publicly available datasets
- A description of any restrictions on data availability
- For clinical datasets or third party data, please ensure that the statement adheres to our policy

The dataset supporting the conclusions of this article is included within the article and its supplementary files. Genome sequence data of CoNS isolates have been deposited in the NCBI Sequence Read Archive under BioProject accession number PRJNA870509. BioSample accession numbers for each genome are listed in Supplementary Table 1.

Human research participants

Policy information about studies involving human research participants and Sex and Gender in Research.

Reporting on sex and gender	n/a
Population characteristics	n/a
Recruitment	n/a
Ethics oversight	n/a

Note that full information on the approval of the study protocol must also be provided in the manuscript.

Field-specific reporting

Please select the one below that is the best fit for your research. If you are not sure, read the appropriate sections before making your selection.

☒ Life sciences ☐ Behavioural & social sciences ☐ Ecological, evolutionary & environmental sciences

For a reference copy of the document with all sections, see nature.com/documents/nr-reporting-summary-flat.pdf

Life sciences study design

All studies must disclose on these points even when the disclosure is negative.

Sample size	Sample size is based on the number of bacterial isolates from clinical animal specimens received by NHVDL. The number of genomes analyzed is based on the number of high quality sequences obtained.
Data exclusions	No data were excluded from the analyses.
Replication	Replication is not relevant because all genomes that were sequenced were included in the analyses.
Randomization	Randomization is not relevant because all genomes that were sequenced were included in the analyses.
Blinding	Blinding is not relevant because all bacterial isolates and genomes were included in the analyses.

Reporting for specific materials, systems and methods

We require information from authors about some types of materials, experimental systems and methods used in many studies. Here, indicate whether each material, system or method listed is relevant to your study. If you are not sure if a list item applies to your research, read the appropriate section before selecting a response.

Materials & experimental systems

n/a	Involved in the study	
☒	☐	Antibodies
☒	☐	Eukaryotic cell lines
☒	☐	Palaeontology and archaeology
☐	☒	Animals and other organisms
☒	☐	Clinical data
☒	☐	Dual use research of concern

Methods

n/a	Involved in the study	
☒	☐	ChIP-seq
☒	☐	Flow cytometry
☒	☐	MRI-based neuroimaging

Animals and other research organisms

Policy information about studies involving animals; ARRIVE guidelines recommended for reporting animal research, and Sex and Gender in Research

Laboratory animals	The study did not involve laboratory animals.
Wild animals	The study did not involve wild animals.
Reporting on sex	Only bacteria from animals were analyzed in this study. Sex not applicable.
Field-collected samples	The study did not include animals collected from the field.
Ethics oversight	All isolates were from animals with confirmed clinical infections. Although the bacterial isolates came from animals, no ethical approval was needed because the bacterial isolates were received by NHVDL from routine clinical specimen submissions from veterinary practices to NHVDL. No live vertebrates were used in this study; hence, the NHVDL was exempt from the International Animal Care and Use Committee (IACUC) approval process as determined at the NHVDL and University of New Hampshire.

Note that full information on the approval of the study protocol must also be provided in the manuscript.
